# Control of V(D)J Recombination through Transcriptional Elongation and Changes in Locus Chromatin Structure and Nuclear Organization

**DOI:** 10.4061/2011/970968

**Published:** 2011-09-29

**Authors:** Beatriz del Blanco, Vanina García, Alberto García-Mariscal, Cristina Hernández-Munain

**Affiliations:** Instituto de Parasitología y Biomedicina López-Neyra (IPBLN-CSIC), Consejo Superior de Investigaciones Científicas, Parque Tecnológico de Ciencias de la Salud, Avenida del Conocimiento s/n. 18100 Armilla, Spain

## Abstract

V(D)J recombination is the assembly of gene segments at the antigen receptor loci to generate antigen receptor diversity in T and B lymphocytes. This process is regulated, according to defined developmental programs, by the action of a single specific recombinase complex formed by the recombination antigen gene (RAG-1/2) proteins that are expressed in immature lymphocytes. V(D)J recombination is strictly controlled by RAG-1/2 accessibility to specific recombination signal sequences in chromatin at several levels: cellular lineage, temporal regulation, gene segment order, and allelic exclusion. DNA cleavage by RAG-1/2 is regulated by the chromatin structure, transcriptional elongation, and three-dimensional architecture and position of the antigen receptor loci in the nucleus. Cis-elements specifically direct transcription and V(D)J recombination at these loci through interactions with transacting factors that form molecular machines that mediate a sequence of structural events. These events open chromatin to activate transcriptional elongation and to permit the access of RAG-1/2 to their recombination signal sequences to drive the juxtaposition of the V, D, and J segments and the recombination reaction itself. This chapter summarizes the advances in this area and the important role of the structure and position of antigen receptor loci within the nucleus to control this process.

## 1. Introduction

**Figure 1:**
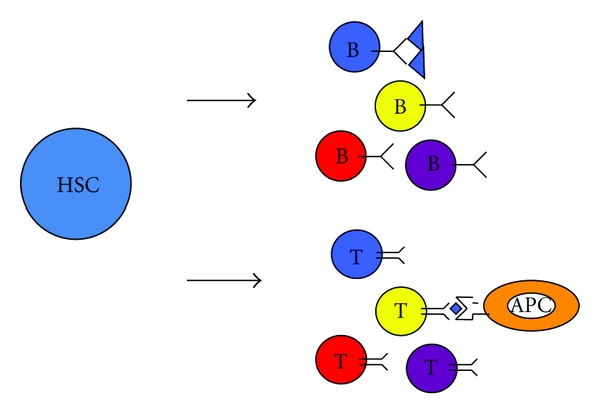
Lymphocyte maturation and expression of clonotypic antigen receptors. Scheme represents lymphocyte maturation from hematopoietic stem cells (HSCs). Each lymphocyte expresses a specific antigen receptor during cell development. B lymphocyte antigen receptors and immunoglobulins (Ig) recognize soluble antigens, whereas T lymphocyte antigen receptors (TCRs) recognize antigenic peptides presented by antigen presenting cells (APCs).

The immune system is considered one of the best models to study the molecular mechanisms of epigenetic control of cellular differentiation *in vivo*. Development of B and T lymphocytes occurs through a series of well-defined differentiation stages initiating from a common hematopoietic stem cell. Each of these differentiation stages involves the activation and repression of antigen receptor loci that influence cellular identity. Expression of these loci in immature lymphocytes requires the activation and silencing of genomic DNA rearrangements that are regulated through the accessibility of chromatin, transcriptional elongation, three-dimensional structure, and nuclear positioning during cell development [1, 2]. In fact, lymphocytes are the only vertebrate cells that use genomic DNA rearrangements as an integral component of their developmental program. This DNA rearrangement process is known as V(D)J recombination and consists of the assembly of the genomically dispersed gene segments V (variable), D (diversity), and J (joining) to generate the functional variable region of antigen receptors: immunoglobulins (Igs) in B lymphocytes and T-cell receptors (TCRs) in T lymphocytes (Figure 1). This process results in the expression of a unique antigen receptor in each developed lymphocyte and is therefore responsible for the generation of antigen receptor diversity in T and B lymphocytes that defines the vertebrate adaptive immune responses. 

**Figure 2:**
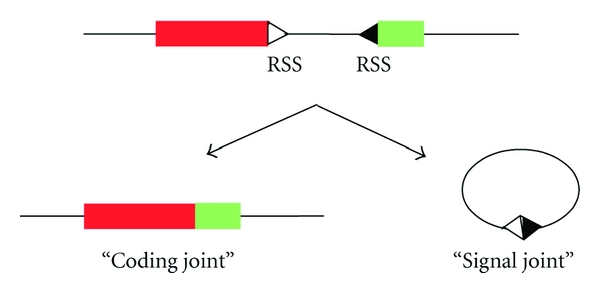
V(D)J recombination process. Gene segments are represented as red and green rectangles, and a pair of compatible RSSs is represented as white and black triangles.

V(D)J recombination is initiated through the action of the protein products of the recombination activating gene (RAG) 1 and 2; together, RAG-1 and RAG-2 form a specific endonuclease in immature lymphoid cells. The RAG-1/2 complex is responsible for the double-strand DNA cleavage between the segments that allows recombination through the recognition of specific recombination signal sequences (RSSs) that flank them (Figure 2); these RSSs consist of conserved heptamer and nonamer elements that are separated by a less conserved spacer region of 12 or 23 base pairs. The 12–23 rule limits recombination to segments between a 12-bp RSS and a 23-bp RSS (here, we will refer to RSSs with 12-bp and 23-bp spacers as compatible RSSs) [3]. Proteins responsible for the joining of the nonhomologous ends then process the double-strand broken ends to generate the coding DNA and the extrachromosomal circles containing the RSSs and the deleted internal gene regions. 

## 2. V(D)J Recombination Control by Chromatin Structure

**Figure 3:**
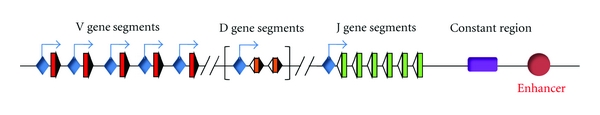
Representation of an antigen receptor locus. V, D, and J gene segments are represented as red, orange, and green rectangles, respectively. RSSs are represented as white and black triangles. The constant region is represented as a purple rectangle. Promoters are represented as blue diamonds, and arrows indicate transcription. The enhancer is represented as a red circle.

**Figure 4:**
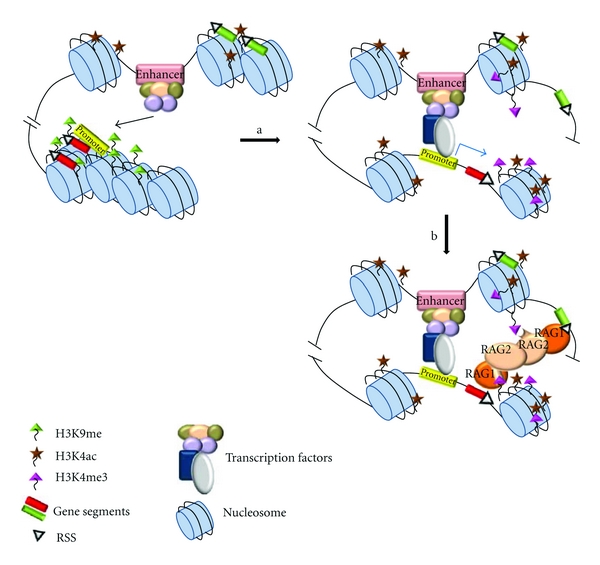
Representation of the molecular mechanism for activation of V(D)J recombination by enhancers, promoters, and transcription. Shown is a depiction of the physical interaction between the enhancer and the promoter within an antigen receptor locus. This interaction is mediated through protein-protein interactions among transcription factors and triggers the processes of transcription and V(D)J recombination that is derived from chromatin opening and subsequent accessibility of the RSSs to the RNAPII and RAG-1/2 proteins. Gene segments are represented as red and green rectangles and RSSs as white triangles. (a) Enhancer activation by the assembly of a functional multiprotein complex on the enhancer mediates the recruitment of the RNAPII to the promoter. This activates germline transcription (blue arrow) and opens the chromatin structure by repositioning nucleosomes, evicting nucleosomes, and/or changing the covalent modifications of histones (e.g., changing H3K9me, which is indicative of repressive chromatin, to H3K4me and H3/H4ac, which are indicative of activated chromatin). (b) The new chromatin configuration allows recruitment of the RAG-2 protein through H3K4me3 and recruitment of the RAG-1 protein to accessible RSSs. Recruitment of a RAG-1/2 complex to two compatible RSSs allows initiation of V(D)J recombination.

The 5′ region of the *Ig *and *Tcr* loci (which encode the variable regions responsible for antigen recognition) contains the V, D (only in some loci), and J gene segments (Figure 3) that are assembled through the action of RAG-1/2 proteins in lymphocyte precursors. The restricted expression of RAG-1 and RAG-2 in immature lymphocytes explains the specificity of the V(D)J recombination process in these cells. However, antigen receptor loci (*Igh, Igk, Igl, Tcra, Tcrb, Tcrg, or Tcrd*) and lineage (B or T) specific regulation is defined by controlled RAG-1/2 accessibility to the specific locus chromatin in B and T lymphocytes precursors [6]. This control establishes that the immunoglobulin loci (*Igh, Igk,* and *Igl*) only rearrange in B-lymphocyte precursors, and the T-cell receptor loci (*Tcra, Tcrb, Tcrd,* and *Tcrg*) rearrange exclusively in T lymphocyte precursors. Additionally, there is temporal control of V(D)J recombination that ensures that this process occurs in a developmental stage-specific manner during lymphocyte development. During T-lymphocyte development, *Tcrb, Tcrd,* and *Tcrg* loci rearrange earlier than *Tcra* locus. Similarly, during B lymphocyte development, *Igh* rearranges earlier than the *Igk* and *Igl* loci. Furthermore, there is an additional developmental control imposed on sets of gene segments within each antigen receptor locus. For example, D-to-J rearrangements precede V-to-DJ rearrangements at the *Tcrb* and *Igh* loci. This locus-, lineage-, temporal-, and gene segment order-specific regulation of V(D)J recombination is mediated through the control of RSS accessibility to the RAG-1/2 proteins. Hence, the chromatin imposes a barrier to RAG-1/2 accessibility that is controlled through strict epigenetic control, which is dependent on the specific antigen receptor locus, gene segment, cellular lineage, and developmental stage. This is the basis for the accessibility model proposed 25 years ago by Yancopoulos and Alt [7]. These investigators observed that the developmental activation of V_H_ gene segment recombination at the *Igh* locus coincided with V_H_ germline transcription (the process of transcription of sterile transcripts at an un-rearranged locus originating from V-associated promoters) during B lymphocyte development [7]. Based on these results, they proposed that the transcription of the V_H_ gene segments reflects an increase in the accessibility of the V_H_ gene segments to both the transcriptional and recombinational machineries (RNA polymerase II (RNAPII) and RAG-1/2 proteins, resp.). Since then, germline transcripts initiating at V, D, and J gene segments have been found to developmentally coincide with the activation of V(D)J recombination at each antigen receptor locus [7–9]. In addition to reports of sense transcription, developmentally regulated antisense intergenic transcription across the V_H_ gene segments that correlates with V_H_ to DJ_H_ recombination has also been reported [10]. In agreement with this model, it has been proven that the barrier that the chromatin imposes on RAG-1/2 accessibility is eliminated through the activation of cis-transcriptional elements present at these loci during lymphocyte development [1]. Each *Ig* or *Tcr* locus is equipped with at least one transcriptional enhancer in the vicinity of the constant region and numerous promoters associated with V, D, and J gene segments (Figure 3). The essential role of each of these cis-elements in controlling the accessibility to the RAG-1/2 proteins was demonstrated in numerous studies using transgenic mini-loci as recombination reporters and directed mutagenesis at the endogenous loci [1]. These studies clearly established that the enhancers are the elements that are responsible for specific lineage determination and temporal control of V(D)J recombination through the general regulation of locus chromatin structure; thus, enhancers control the accessibility of the RAG-1/2 proteins to multiple gene segments separated by large distances, whereas promoters are the elements that mediate the accessibility of the RAG-1/2 proteins to regions located at the proximal regions of the specific gene segments [1]. The accessibility model was reinforced by observations demonstrating a direct correlation between V(D)J recombination and activating epigenetic modifications such as histone H3 and H4 acetylation (H3ac and H4ac), methylation of lysine 4 of histone H3 (H3K4me), nuclease accessibility and DNA hypomethylation [1, 11–14], and changes in nucleosomal structure [15]. Furthermore, establishment of inactive chromatin suppresses V(D)J recombination [16]. Additional *in vitro* studies have demonstrated that assembly of RSSs into nucleosomes inhibits V(D)J recombination [17–20], supporting the notion that nucleosomes impede RAG1/2 binding or function. The barrier for V(D)J recombination imposed by nucleosomes can be surmounted by ATP-dependent chromatin remodeling complexes, such as SWI/SNF [17, 18, 21–23]. Recently, it has been directly demonstrated that chromatin accessibility to RAG-1/2 is indeed mediated by enhancers and promoters [24]. In this study, it was proven that the enhancers control global RAG-1 binding, whereas promoters direct local RAG-1 binding at the antigen receptor loci. RAG-1 binding to accessible RSSs can be targeted in the absence of RAG-2, which is recruited directly to trimethylated H3K4 (H3K4me3), a mark of open and active chromatin [25–27]. Hence, both the enhancer and promoter elements are necessary to confer RAG-1/2 accessibility to specific RSSs within a given locus and to facilitate the recombination synapse between the RSSs (Figure 4). To date, the precise molecular mechanisms by which distal enhancers control the transcriptional activation from promoters separated by large distances and V(D)J recombination are not known. Chromatin immunoprecipitation and chromosomal conformation capture experiments have demonstrated that V(D)J recombination involves physical interactions between distal transcriptional regulatory elements such as promoters and enhancers to permit RAG-1/2 accessibility to their target RSSs [28–30]. 

It is interesting that the antigen receptor loci undergo a process of gene contraction (juxtaposition of V and D-J regions), which is strictly regulated during development. As demonstrated by three-dimensional fluorescence *in situ* hybridization (3D-FISH) experiments using distal DNA probes, this contraction correlates with transcription and V(D)J recombination [29, 31–33]. In fact, gene contraction occurs at the same moment that a particular locus is transcribed and is ready to recombine even in the absence of recombinase activity in *Rag* ^−/−^ mice. These contractions could be mediated by interactions between regulatory regions and/or by specific nuclear structures. Enhancer-promoter interactions are thought to direct the long-distance communications (Figure 4). Comparative analysis of *Tcra/Tcrd* locus contraction in wild-type, *Tcra* locus enhancer (E*α*)^−/−^ and *Tcrd* locus enhancer (E*δ*)^−/−^ thymocytes revealed no significant differences between the cell types [33]. Hence, enhancer-promoter interaction is not necessarily sufficient to mediate *Tcra* locus contraction and RSSs synapse, but locus contraction might facilitate both enhancer-promoter interactions and RSSs synapse that are required for transcription and V(D)J recombination. These data suggest that a preexisting conformation of the locus that is mediated by an enhancer-independent mechanism may promote the enhancer-promoter interactions necessary to activate transcription and V(D)J recombination of the distant gene segments [32, 33]. Hence, enhancer-promoter interactions could establish molecular bridges over long distances but only after they have been brought into proximity by locus contraction mediated by chromatin-organizing proteins. The mechanism for locus contraction itself is not known. Deficiencies in specific transcription factors such as Pax5, Ikaros, and YY1 disrupt the contracted *Igh* locus configuration, but it is not known how they do this [34–38]. Additionally, locus contraction could be mediated by chromatin-organizing proteins such as SATB1, CCCTC-binding factor (CTCF), and CTCF-associated cohesin that have been shown to promote long-distance looping interactions at other loci [39–44]. In fact, CTCF and cohesin have been shown to colocalize at multiple sites within the *Igh* and *Igk* loci in immature B lymphocytes [45, 46]. Consistent with this, very recent experiments have functionally demonstrated that CTCF and cohesin influence the genomic structure of the *Igh* locus in developing B lymphocytes [47]. The precise molecular mechanisms involved in how locus-specific conformational changes can regulate the enhancer-promoter interactions to subsequently direct the different V(D)J programs during lymphocyte development is an issue of intense research in the field. 

## 3. V(D)J Recombination Control by Transcriptional Elongation

The finding that germline transcription at a given antigen receptor locus occurs concomitantly with its recombination [7] suggests a linkage between transcription and V(D)J recombination. Based on this evidence, it is accepted that both transcription and V(D)J recombination are consequences of locus accessibility that is mediated through the activation of promoters and enhancers during lymphocyte development [1]. These regulatory elements serve as docking elements to recruit transcription factors that initiate and help to propagate changes in chromatin structure that are essential for the accessibility of the RNAPII and RAG-1/2 proteins (Figure 4). Consistent with this, several transcription factors have also been shown to coordinately regulate both transcription and V(D)J recombination. For example, overexpression of E2A in nonlymphoid cells that express RAG-1 and RAG-2 proteins induced germline transcription and V(D)J recombination at the *Igk*, *Tcrg,* and *Tcrd* loci [48, 49]; OcaB^−/−^ mice displayed defective transcription and recombination of a subset of V*κ* gene segments [50]; Stat5 is required for the transcription and V*γ*J*γ* recombination at the *Tcrg* locus in response to IL-7 [51, 52]; and deletion of the enhancers and promoters at *Tcr* and *Ig* loci or inclusion of mutations at motifs for required transcription factors within these cis-elements inhibit both transcription and V(D)J recombination at each locus [1]. 

**Figure 5:**
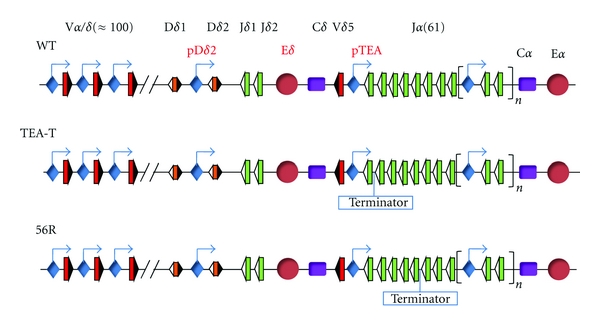
Representation of WT *Tcra* locus and mutant versions containing a terminator sequence [4, 5]. V, D, and J gene segments are represented as red, orange, and green rectangles, respectively. RSSs are represented as white and black triangles. Constant regions are represented as purple rectangles. Promoters, including the TEA promoter, are represented as blue diamonds, and arrows indicate transcription. E*δ* and E*α* are represented as red circles. The position of the terminator sequence in mutant TEA-T and 56R *Tcra* is indicated.

For many years, it was not clear whether germline transcription was merely a side effect of chromatin accessibility generated by the activation of enhancers and promoters or whether it was causal in the V(D)J recombination process itself. In an elegant and definitive study, Abarrategui and Krangel have proven that germline transcription is a key developmental regulator of accessibility for V*α*-to-J*α* recombination at the *Tcra* locus [4]. This locus spans 1.5 Mb and contains around 100 V*α/δ* gene segments in a 1 Mb region at the 5′ end of the locus and 61 J*α* gene segments in a 65 kb region at the 3′ end of the locus [53] (Figure 5). V*α*-to-J*α* recombination events depend on both E*α*, located at the 3′ end of the locus [54], and the promoters associated with the J*α* gene segments [55]. These investigators introduced a strong transcription terminator downstream of either the TEA promoter (TEA-T) or the J*α*56 gene segment (56R) in the endogenous mouse locus to block transcription originating at the upstream TEA promoters [4, 5] (Figure 5). The terminator sequence they used consists of four polyadenylation sites followed by an array of twelve bacterial lac operons that are thought to function as strong pause sites for RNAPII. The introduced terminator was able to impose an effective block to RNAPII passage. Interestingly, the transcriptional block in both TEA-T and 56R mice caused a strong reduction in recombination at the J*α* gene segments located immediately downstream of the terminator sequence. Thus, these experiments clearly demonstrated that transcriptional elongation by RNAPII is necessary for creating accessible chromatin for the RAG-1/2 proteins to initiate *Tcra* recombination. 

Although instances of recombination in the apparent absence of transcription have also been reported, credible explanations for each of them can be found. For example, isolated nuclei of lymphocytes from *Rag* ^−/−^ mice can rearrange their receptor antigen loci *in vitro* by addition of RAG-1 and RAG-2 proteins in the absence of ongoing transcription [6]; however, the chromatin structure of the receptor antigen loci could remain accessible during nuclei isolation. Additionally, it has been demonstrated that the V_H_ gene segments rearrange in pro-B lymphocytes with no detectable transcription of these segments [56]; however, the state of transcription of these segments at the time of recombination could not be analyzed. Furthermore, the requirement for transcription itself might not be necessary for RSSs that are located near a cis-element; this is the case for RSSs that are positioned adjacent to a promoter. Several examples of this phenomenon include the inducible mouse mammary tumor virus long terminal repeat that can confer accessibility to a tightly associated RSS [57], the endogenous *Tcrb* locus that has a *Tcrb* enhancer-(E*β*-) dependent promoter tightly associated with the D*β*1 segment to confer accessibility to this segment [58, 59], and the physical interaction of the D*β*1 promoter with E*β* to deliver SWI/SNF chromatin remodeling complexes that results in a decrease of nucleosome occupancy at the D*β*1 gene segment [28, 30, 60]. These results argue that transcription is not required to confer accessibility to RAG-1/2 when the RSS is tightly associated with a promoter. However, this is not the situation for many RSSs within the antigen receptor loci *in vivo* because they are positioned far away from the promoters. 

More recent experiments with the TEA-T and 56R mouse models have definitively demonstrated that chromatin accessibility to the RAG-1 protein is mediated by transcriptional elongation itself [24]. In this study, it was proven that the transcriptional terminator introduced within the J*α* cluster in TEA-T and 56R mice (Figure 5) inhibited the recruitment of the RAG-1 protein to downstream chromatin. Hence, transcriptional elongation itself confers accessibility to the RAG-1/2 proteins to specific RSSs. Although the pattern of RAG-2 binding was not assessed in this study, it is expected that it would closely resemble that of RAG-1 binding because the pattern of H3K4me3 (which accurately predicts RAG-2 binding) was similar to that of RAG-1 [4, 5, 24, 27]. Hence, H3K4me3 recruits the RAG-2 protein [25–27] and directly contributes to the opening of the chromatin by either repositioning or evicting nucleosomes, which allows free access of the RAG-1 protein to RSSs. Although it has not been directly demonstrated, it is expected that this is likely to be the case for the RSSs that are distantly located from the promoters in the other *Ig* and *Tcr* loci. However, a direct role for transcripts in directing V(D)J recombination, as has been shown for class switching at the *Igh* locus, is not expected because the RAG-1/2 substrate is double strand DNA. 

Thus, Abarrategui and Krangel's studies [4, 5] have demonstrated that V(D)J recombination at the *Tcra* locus requires that the elongation machinery travels or has traveled through the RSS's DNA to allow the RAG-1/2 proteins to access to the RSS's chromatin. However, the precise molecular mechanism by which transcriptional elongation directs the process of V*α*J*α* recombination is not known. Chromatin structure imposes significant obstacles on the passage of the RNAPII through the DNA. Because the elongation of transcription is associated with the transient disruption of nucleosome structure [61] and because of the transcription-dependent reduction of nucleosome density at the coding region [62], it is possible that RAG-1 is recruited to the RSSs due to either a transient disruption of the chromatin structure or to less compacted chromatin derived from RNAPII transit. Consistent with the role of transcriptional elongation in transient disrupting of nucleosomal structure, it has been shown using the TEA-T mouse model (Figure 5) that germline transcription originating from the TEA promoter at the *Tcra* locus causes covalent histone modifications related to opening of chromatin [5], as well as repositioning and loss of the nucleosomes at a 600 base-pair region including the TEA promoter itself and its closest 3′ J*α* gene segment, J*α*61 [15]. 

Gene transcription is initiated by the binding of transcription factors to promoters and enhancers (Figure 4). The binding of transcription factors to the enhancer recruits histone acetyltransferases (HATs) that acetylate the N-terminal tails of histones H3 and H4. These acetylated histones provide binding sites for the bromodomains present in other chromatin remodeling complexes and histone-modifying enzymes. Binding of these complexes and enzymes results in nucleosome displacement or disassembly and thus frees promoters for binding by RNAPII thereby allowing transcriptional initiation [63–65]. The RNAPII complexes assembled on the promoter subsequently transit through the chromatin to mediate transcriptional elongation. Clearance of the RNAPII from the promoter requires the phosphorylation of its carboxy-terminal domain (CTD) [66], which is a molecular platform that can recruit a variety of histone modifier complexes, chromatin remodeling complexes, histone chaperones, and elongation factors that are associated and travel with the elongating form of RNAPII; these cofactors are required for efficient transcription through the chromatin [65, 67–72]. Among the most important chromatin modifications associated with transcriptional elongation are the methylation of lysines 4, 36, and 79 of histone H3 and the monoubiquitination of histone H2A. 

Abarrategui and Krangel observed that H3ac, H3K4me3, H3K4me2, and H3K36me3 were significantly reduced at the 3′ end of the terminator introduced immediately downstream of the TEA promoter at the *Tcra* locus [5] (Figure 5); these results suggest that the histone methyl transferases and HATs responsible for these histone covalent modifications might be involved in activating V*α*J*α* recombination through RAG-1/2 recruitment mediated by transcriptional elongation [70, 73]. In addition, H3K4me2/3 that recruits RAG-2 [25–27] also recruits other chromatin remodeling complexes such as the ATPase RSC, ISWI, or SWI/SNF that can reposition or evict nucleosomes to facilitate the passage of the RNAPII that can be involved in recruitment of RAG-1 [23, 74–76]. Currently, the identity of the specific chromatin modifying activities that are involved in facilitating the recruitment of RAG-1/2 proteins to RSSs is unknown. Additionally, chaperones that travel with the elongating RNAPII and the chromatin remodeling complexes that facilitate RNAPII's transit could also favor RAG-1 binding [61, 77]. It is also possible that RAG-1 recruitment to the RSSs might be facilitated by direct interaction with the elongating RNAPII through the RNAPII CTD. This would require a total coupling between transcription and V(D)J recombination; the coupling of transcription and RNA splicing is already accepted to occur at the RNAPII CTD [78]. Understanding how precisely transcription activates V(D)J recombination is an important goal for future research in this field.

## 4. Control of V(D)J Recombination by the Nuclear Position of the Antigen Receptor Loci

In spite of the above cited data, there is evidence that shows that transcription and RSS accessibility are not necessarily sufficient to activate V(D)J recombination *in vivo*. For example, in pre-T lymphocytes, a deletion within the *Tcrb *locus that placed the V*β* gene segments under the influence of the E*β* promoted high levels of V*β* transcription but not V*β* to DJ*β* rearrangement [79]. Additionally, in pre-T lymphocytes, ectopic introduction of E*α* within the cluster of V*β* gene segments enhanced transcription of these segments but did not induce V*β*-to-DJ*β* rearrangement [80]. Furthermore, germline V*β* transcripts are detected from both alleles of the *Tcrb* locus even in the presence of allelic exclusion during V*β*-to D*β*J*β* recombination [81, 82]. Finally, germline transcription similarly occurs at the *Igk* locus, which undergoes bi-allelic germline transcription in pre-B lymphocytes during allelic exclusion of V*κ*-to-J*κ* recombination [83–86]. These findings indicate that additional regulatory constraints on V(D)J recombination exist that operate beyond transcription and chromatin accessibility. 

Over the last ten years, it has become clear that the position of *Ig* and *Tcr* loci in the nucleus has an essential role in directing V(D)J recombination between distantly located gene segments. It is now accepted that, in addition to transcriptional competence, a particular locus or allele must move away from repressive chromatin to allow distant RSSs to form a recombinational synapse through RAG-1/2 binding that initiates V(D)J recombination [87]. It is now evident that the *Ig *and *Tcr* loci move away from repressive compartments such as the nuclear periphery or pericentric heterochromatin when they undergo recombination; the loci then reassociate with them following recombination [85, 87]. Furthermore, associations of *Igh*, *Igk,* and *Tcrb* loci with repressive nuclear compartments seem to be responsible for the establishment of allelic exclusion [81, 86, 88]. Hence, there is a clear connection between the association with repressive nuclear compartments and the inhibition of V(D)J recombination, but germline transcription does not always correlate with this phenomenon. 

 The mechanism by which locus association with repressive compartments inhibits V(D)J recombination without inhibiting transcription remains unknown. RAG-1/2 binding to D and J gene segments is robust at the *Igh *and *Tcrb* loci in pro-B/pre-B and pro-T/pre-T lymphocytes, respectively, even when V-to-DJ recombination is inhibited and one or both alleles are associated with repressive compartments in both stages [24, 27, 31, 81]. It is known that the *Igh* locus is tethered to the nuclear membrane through the distal V_H_ region cluster, whereas the D_H_J_H_ region is located away [85]. Thus, persistent RAG-1/2 binding to *Igh* and *Tcrb* D and J gene segments in pro-B/T and pre-B/T lymphocytes might be consequence of RSS accessibility due to the spatial orientation of these loci within the nucleus [27, 85]. It is known that V_H_ and V*β* gene segment transcription and accessibility are reduced in the transition from pro-B/T to pre-B/T lymphocytes, respectively, and hence both parameters do seem to correlate with allelic exclusion of *Igh* and *Tcrb* loci at pre-B/T lymphocytes [89–91]. These results support the current model that feedback inhibition of *Igh* and *Tcrb* loci in pre-B/T lymphocytes, but not in pro-B/T lymphocytes, operates primarily on V_H_ and V*β* gene segment accessibility, respectively. The different chromatin structure at the V_H_ and V*β* gene segments in the two stages indicates clear differences about what might be happening in pro-B/T versus pre-B/T lymphocytes [27, 81, 89–91]. Identification of cis-elements and transfactors that are involved in controlling the association of specific antigen receptor alleles with repressive nuclear compartments is required to elucidate the function of nuclear positioning in regulating V(D)J recombination and allelic exclusion. In the case of the *Igk* locus, which is also relocated during B lymphocyte development [85], a cis-element that binds the Ikaros transcriptional repressor targets *Igk* transgenes to centromeric chromatin and inhibits V*κ*-to-J*κ* recombination [92]; another candidate is IRF-4, which directs the *Igk* allele away from the pericentromeric heterochromatin [93]. In the case of the *Tcrb* locus, the helix-loop-helix protein, E47, is a good candidate to direct the interaction of this locus with pericentromeric heterochromatin since its dosage is rate-limiting with regard to V(D)J recombination and forced E47 expression interferes with pre-TCR-mediated feedback inhibition [94]. Additionally, it was also proposed that V(D)J recombination events occurring on one allele could activate signals that inhibit rearrangements on the second allele [95]. Consistent with this idea, it has been shown recently that homologous pairing of *Ig *alleles occurs during recombination and is mediated by RAG-1/2 binding [88, 96]. Furthermore, it has been demonstrated that RAG-mediated cleavage on one allele induces the other allele to relocate to pericentromeric heterochromatin by a mechanism related to the recognition of the cleaved allele by the DNA damage sensor ataxia telangiectasia mutated (ATM) protein [88]. Hence, activation of ATM by the cleaved allele acts in trans on the uncleaved allele to prevent recombination. Interallelic pairing has been proposed as a general mechanism for establishing the monoallelic gene expression that contributes to the maintenance of genomic integrity and suppresses oncogenic translocations during V(D)J recombination of antigen receptor loci.

## 5. Conclusions

V(D)J recombination is essential for the development of adaptive immune responses in vertebrates. In developing lymphocytes, V(D)J recombination is subjected to very tight spatial and temporal regulation. The regulation of this process is very complex and involves nuclear dynamics and changes in higher-order chromatin architecture to create gene segment accessibility to RAG-1/2 proteins. Active chromatin is bound by RAG-2 through interactions with specific H3K4me3, whereas RAG-1 binds to accessible RSSs derived from transcriptional elongation in large loci. The correlation between transcription and V(D)J recombination in both the recombined and allelic excluded antigen receptor loci led to the studies that established that the differential positioning of such loci at transcriptionally repressive nuclear regions might be responsible for allelic exclusion. Allelic association with the repressive nuclear compartments can inhibit V(D)J recombination by a mechanism other than transcription or RAG-1/2 accessibility to chromatin. Future experiments should be focused on identifying the cis-elements and tran*s*-factors that regulate V(D)J recombination *in vivo.*
